# Pulling Rank: Military Rank Affects Hormone Levels and Fairness in an Allocation Experiment

**DOI:** 10.3389/fpsyg.2016.01750

**Published:** 2016-11-11

**Authors:** Benjamin Siart, Lena S. Pflüger, Bernard Wallner

**Affiliations:** ^1^Department of Anthropology, University of ViennaVienna, Austria; ^2^Department of Behavioural Biology, University of ViennaVienna, Austria

**Keywords:** hierarchy, status, military rank, cortisol, testosterone, allocation experiment, social

## Abstract

Status within social hierarchies has great effects on the lives of socially organized mammals. Its effects on human behavior and related physiology, however, is relatively little studied. The present study investigated the impact of military rank on fairness and behavior in relation to salivary cortisol (C) and testosterone (T) levels in male soldiers. For this purpose 180 members of the Austrian Armed Forces belonging to two distinct rank groups participated in two variations of a computer-based guard duty allocation experiment. The rank groups were (1) warrant officers (high rank, HR) and (2) enlisted men (low rank, LR). One soldier from each rank group participated in every experiment. At the beginning of the experiment, one participant was assigned to start *standing guard* and the other participant *at rest.* The participant who started *at rest* could choose if and when to relieve his fellow soldier and therefore had control over the experiment. In order to trigger perception of unfair behavior, an additional experiment was conducted which was manipulated by the experimenter. In the manipulated version both soldiers started in the *standing guard* position and were never relieved, believing that their opponent was *at rest*, not relieving them. Our aim was to test whether unfair behavior causes a physiological reaction. Saliva samples for hormone analysis were collected at regular intervals throughout the experiment. We found that in the un-manipulated setup high-ranking soldiers spent less time *standing guard* than lower ranking individuals. Rank was a significant predictor for C but not for T levels during the experiment. C levels in the HR group were higher than in the LR group. C levels were also elevated in the manipulated experiment compared to the un-manipulated experiment, especially in LR. We assume that the elevated C levels in HR were caused by HR feeling their status challenged by the situation of having to negotiate with an individual of lower military rank. This would be in line with the observation that unequally shared duty favored HR in most cases. We conclude that social status, in the form of military rank affects fairness behavior in social interaction and endocrine levels.

## Introduction

Social hierarchies exist in virtually all human societies as well as in many non-human species. In humans hierarchy is defined as an implicit or explicit rank order of individuals or groups with respect to a valued social dimension (Magee and Galinsky, 2008). In evolutionary terms, social hierarchies are thought to bear adaptive advantages. For instance, it has been argued that social hierarchy reduces in-group conflicts by enhancing voluntary cooperation, supports the division of labor, and capitalizes on the complementary psychological effects of having versus lacking power (Halevy et al., 2011). Socio-economic status (SES) is the most commonly used concept to study effects of status in humans. SES is a multidimensional construct comprising diverse socioeconomic factors that is used to conceptualize an individual’s social standing or class (Braveman et al., 2005). The socioeconomic factors most frequently used in SES are occupation, income and education. An important aspect of human social hierarchy is its effect on health (Marmot, 2004). Many negative health symptoms are related to physiological stress reactions in individuals representing low levels of SES (National Center for Health Statistics, 2012; Saydah et al., 2013; Mackenbach et al., 2015). Thereby status has been linked, among other factors, to cardiovascular disease (Winkleby et al., 1992), depression, obesity, and diabetes (Everson et al., 2002), with low-status groups being more vulnerable to such diseases than high-status groups (reviewed in Adler et al., 1993). Childhood SES has been shown to affect brain structure. Studies using structural MRI found prefrontal cortical thickness (Lawson et al., 2013) and brain surface area (Noble et al., 2015) to be positively correlated to higher SES in children and adolescents. Increased stress in low-status individuals is thought to be a major cause of the correlation between low social status and decreased health. The reasons for increased physiological stress in individuals with low social status are considered to be a decreased predictability and control in life, fewer outlets for frustration, and diminished social support (reviewed in Sapolsky, 2004). The steroid hormone cortisol (C), secreted from the adrenal cortex (reviewed in Tsigos and Chrousos, 2002), plays a significant role in mediating stress-related diseases. In humans and in non-human primates the association between C levels and social rank positions are well investigated. In humans, high status was correlated with lower levels of C (Sherman et al., 2012). A recent study found that SES of mothers is correlated with salivary C levels in mother-infant dyads, with low SES being associated with high C levels in both mothers and infants (Clearfield et al., 2014). According to Miller et al. (2009), effects of childhood SES persist well into adult life: persons that belonged to groups with low SES during early life were found to show increased output of C in daily, adult life. An inverse correlation between social rank and C has also been described in several non-human primate species. This was associated to decreased access to resources or lowered social support in subordinates compared to high-ranking individuals (Abbott et al., 2003). Nonetheless, the effect of an individuals’ social rank position within a primate society in relation to stress reactivity can be diverse. For example, other studies reported that higher ranking males are more physiologically stressed than lower ranking individuals (Barrett et al., 2002), particularly during episodes of social instability (Higham et al., 2013). Similar inconsistencies were also found in studies on social rank and C levels in humans (for a review see Dowd et al., 2009).

Besides C, the sex steroid testosterone (T) has received considerable attention in studies on social status of primates including humans (Sapolsky, 1991; Mazur and Booth, 1998; Sherman et al., 2015). T has been argued to influence aggressive and dominant behavior (Mazur and Booth, 1998). In sports, T levels generally rise in anticipation of a competition and are elevated in winners in comparison to losers after the competition (Booth et al., 1989). Apart from aggressive or dominant behavior, T has been argued to affect other aspects of social behavior associated with status seeking, such as honesty and trust. Administration studies have shown, that a single dose of T decreases interpersonal trust in individuals who give trust easily (Bos et al., 2010). This effect is thought to be advantageous in competition for status and resources, where too much trust may be disadvantageous (Bos et al., 2010). A recent study found female poker players to reduce bluffing and increase the calling of suspected bluffs after T administration compared to a placebo (Van Honk et al., 2016). The authors argue that status seeking leads to an increased motivation not to get caught bluffing, as this would damage the players reputation, and to call the other players bluffs because not calling bluffs would seem submissive (Van Honk et al., 2016). In humans most single T administration studies have been performed in females (for a review see Bos et al., 2012). However, evidence from studies on endogenous testosterone indicate, that associations between testosterone and dominance related behavior are sex independent (Harris et al., 1996; Van Honk et al., 1999).

Although T has been studied thoroughly, there is no consensus on the effects of T on human behavior such as aggression (reviewed in Archer, 2006; Montoya et al., 2012), dominance, (Mazur and Booth, 1998), and fairness (Eisenegger et al., 2010; Wright et al., 2012). These discrepancies are thought to be caused in part by the interaction of C and T (reviewed in Carré and Mehta, 2011; Montoya et al., 2012). The dual-hormone hypothesis proposes that high T levels lead to elevated status only in individuals with low C levels (reviewed in Mehta and Prasad, 2015). Multiple studies support that hypothesis. For example, in male human executives, low levels of salivary C in combination with high T levels are related to the number of subordinates an executive officer has (Sherman et al., 2015). A study on female collegiate athletes yielded similar results. Here, status with teammates was positively related to levels of T, but only if these occurred in combination with relatively low levels of C (Edwards and Casto, 2013).

Both social status and endocrine correlates have been investigated in the context of fairness behavior in humans (Ball et al., 2001; Zak et al., 2009; Eisenegger et al., 2010; Piff et al., 2012; Pfattheicher et al., 2014). Findings gained in economic experiments indicate that expectations of fairness in humans vary greatly in respect of their social status: Status apparently modifies the perception of what is fair or acceptable. In bargaining situations, individuals with lower status are more likely to accept being overreached by higher status individuals than vice versa (Ball et al., 2001; Hu et al., 2014, 2016) Low status individuals are also more satisfied with disadvantageous payoffs than individuals with higher status (Albrecht et al., 2013). Van Prooijen et al. (2002) sum it up as follows: people perceive the outcome of an interaction to be fair if they think that they are entitled to that outcome, and social status can determine whether people perceive themselves as entitled to certain outcomes.

A recent study utilizing EEG data of event-related potentials associated with arousal showed that status indeed modulates brain activity in response to unfairness. Event related potentials (late positivity potential, 400–700 ms) after unfair offers were more pronounced in the high status condition compared to the low status condition (Hu et al., 2014). In the same study individuals in the high status condition were less likely to accept unfair offers. FMRI data collected in a similar study yielded similar results (Hu et al., 2016). High status individuals more frequently rejected unfair offers and the effect of status was correlated with the activation of brain areas related to fairness perception.

While correlational studies on the relationship between status and factors such as health and hormonal status utilize measures such as the SES, rank in economic experiments is usually assigned artificially. The advantage of induced rank is that it can be manipulated by the experimenter and is independent of factors such as income, social background, and education. The approach of assigning ranks artificially has been proven to be a practical tool in the laboratory (Ball et al., 2001; Albrecht et al., 2013; Hu et al., 2014). Similar to studies using SES, artificially assigned rank affects physiological stress reactions in experimental setups (Mendelson et al., 2008; Akinola and Mendes, 2013; Gramer and Schön, 2015).

Previous studies using artificial rank, however, leave us with the question to what extent the findings obtained under these conditions can be translated to social hierarchy effects in real life. So far only a few studies used real-life hierarchies to study the connection between status and fairness. A study utilizing the brand and model, age, and appearance of vehicles to indicate the drivers’ social class found upper-class drivers to be more likely to cut off other vehicles at an intersection and pedestrians at a crosswalk, than lower-class drivers (Piff et al., 2012). Another study using SES determined that lower class individuals were more generous, charitable, trusting, and helpful than high SES individuals in four separate experiments (Piff et al., 2010). Research suggests that social status among a group of peers on a so-called local ladder is more important for the individuals than his/her status within a greater society. The in-group concept established by the local ladder was introduced as sociometric status (SMS). SMS focuses on face-to-face groups, such as among neighbors, co-workers, or classmates (Anderson et al., 2001, 2012). Accordingly, a study on police officers showed that status among fellow officers is a better predictor for physiological reactivity during a stress task than status among a greater society, i.e., all citizens of the United States (Akinola and Mendes, 2013).

To summarize, data from real life-studies show effects of status to be especially important in local ladders and to be correlated with physiological stress. Hormones are thought to influence fairness in humans and data obtained mostly from laboratory studies indicate status to influence fairness behavior. Now, there is a need to investigate how hierarchical rank within a real-life local ladder affects fairness in an experimental setup and its interaction with participants’ endocrine reactions. We hypothesized that rank within a social hierarchy would affect fairness behavior and endocrine reactions in response to fair or unfair behavior. In order to test our hypothesis we investigated members of the Austrian Armed Forces and exposed them to a newly designed allocation experiment that did not use money as an incentive. We chose this cohort because the military is a perfect example of a strict real-life social hierarchy. In the allocation experiment, two participating soldiers of different military rank were confronted with the task to divide up an unwelcome, but metabolically not costly, military duty, i.e., being at guard duty instead of being at rest during a 40 min computer-based interaction. By choosing a task that was unwelcome but not in itself stressful, we investigated whether unfair allocation of the duty would cause an endocrine reaction in low- and/or high-ranking soldiers. More precisely we investigated whether (i) the military rank of the participants is associated with T and C levels or the interaction of both steroids before and after the experiment, (ii) the military rank affects the outcome of the experiment (how the 40min are divided), and (iii) whether unfair outcome of the experiment correlates with endocrine status, and (iv) whether military rank is a predictor for the severity of that effect.

## Materials and Methods

### Participants and Study Sites

Studies were conducted in six military barracks; four are located in Lower Austria (Großmittel, Mistelbach, Zwölfaxing, and Horn), one in Upper Austria (Linz-Hörsching), and one in Burgenland (Güssing). The experiments took place between December 2013 and February 2015 in crew accommodation rooms or recreational rooms (e.g., staffrooms or tea-kitchens) at the above mentioned barracks.

The Austrian ministry of defense stipulated that only volunteers were allowed to participate in this study. The army’s internal ethics commission gave permission to all proceedings in this study. Participants were either warrant officers (equivalent to ranks ranging from OR-5 to OR-9 on the NATO standard rank scale) or enlisted men (equivalent to ranks ranging from OR-1 to OR-4 on the NATO standard rank scale) of the Austrian Armed Forces. These soldiers are particularly well suited for our investigation because (i) they are part of a large cohort with a strict predefined hierarchy, (ii) their rank is recognizable by badges of rank and therefore, immediately recognizable, (iii) in a military environment all cohort peers are aware of the importance of the local hierarchy ladder, and (iv) individual differences in income and education within the whole study population were minimal (see below).

Before participating in the study each soldier gave written informed consent to all procedures in the study. Participants were informed about the duration of the allocation experiment (40-min computer interaction) followed by a 45-min cooldown phase. Furthermore, each volunteer was asked to provide saliva samples for hormone analyses at regular time intervals during the experiment (see Collection and Analyses of Saliva Samples). The procedures of the allocation experiment were explained to them via a rules sheet directly before the start of the 40-min computer interaction.

In total, 180 male members of the Austrian Armed Forces volunteered, comprising 90 warrant officers (mean age = 34.1 ± 10.6 ± SD years) and 90 enlisted men (mean age = 22.2 ± 2.4 ± SD years). Enlisted men and warrant officers were chosen because they are part of the same career-path. Enlisted men can be promoted to warrant officer ranks and every warrant officer started his career as an enlisted man. In contrast, Austrian commissioned officers follow a different career path including three years of academic studies at the military academy. In this study, we refer to high rank (HR) for participants having the rank of warrant officer and to low ranks (LR) for enlisted men. Highest educational attainment was very similar in HR and LR. In LR, 47.7% of participants finished an apprenticeship; for HR that number is 50%. 21.6% of LR and 18.2% HR had a high school diploma; for 15.9% of each group, mandatory school was the highest educational attainment. One member of each group studied at a university.

### Experimental Design

Experimental sessions began between 09.50 AM and 11.20 AM on workdays. This 90 min time frame was chosen in order to reduce bias in hormone levels caused by circadian changes and to minimize interference with the soldiers’ military duties. Given the soldiers daily routines, holding experimental sessions later than that would not have been feasible.

In each experimental session (manipulated or un-manipulated; see Sections “Un-manipulated Setup” and “Manipulated Setup”), two soldiers of differing military rank, a HR and a LR, participated. Participants in each session were working at the same barracks but were not part of the same military unit, thus not working together directly. This reduced the likelihood of personal matters influencing the experiment. In contrast to classic economic experiments, our allocation experiment did not involve money as an incentive. Our design emulated a situation where 40 min of guard duty have to be shared between two soldiers. Soldiers in the experiment could be in one of two positions: *standing guard* or being *at rest*. *Standing guard* consisted of having to stand in front of a computer, guarding it. Soldiers in the *at rest* position were allowed to sit in front of the computer while the screen showed images of landscapes or plants. A participant could only change from the *standing guard* position to the *at rest* position if he was informed via the computer that the other soldier relieved him. Importantly, a participant in the *at rest* position was free to choose not to relieve his opponent at all. This setup confronted the participants with the task to divide up a not very unpleasant but nevertheless unwelcome duty, in this case *standing guard*. There were two types of setups for the experiment, one un-manipulated and one manipulated setup, both sharing a common introductory phase.

#### Introductory Phase

After both participants arrived at the site of the experiment, they were provided with instructions about the procedure of saliva sampling during the experiment. In this phase the participants were able to see each other and thereby recognize each other’s military rank. Military uniforms, including badges of rank, were worn throughout the experiment. After the introductory phase, participants were placed in separate rooms, where they were asked to play an allocation experiment with their opponent via connected computers. The rooms were equipped with a chair and a table. On the table was a computer screen, a computer mouse, and a sheet of instruction for the allocation experiment were placed along with the kit for the saliva samples, including a jar filled with ice cubes to store the collected samples. Participants were asked to carefully read the instructions before starting the experiment by clicking a start-button. In the instructions the participants were informed that they have to play a game in which they have to negotiate about 40 min of “Guard Duty” (see Sections “Experiment 1 – Un-manipulated Setup” and “Experiment 2 – Manipulated Setup”). Additionally, they were informed that messages on the screen would show up at regular intervals, requesting them to provide saliva samples. As soon as both soldiers activated the start button either the un-manipulated or the manipulated setup started. Both setups were programmed using z-tree, a software package for economic experiments (Fischbacher, 2007).

#### Un-manipulated Setup

In the un-manipulated setup, participants interacted with each other without any intervention of the experimenter. One soldier A had to start *standing guard*, while the other soldier B started the experiment *at rest*. Whether a particular soldier started the experiment *at rest* or *standing guard* was assigned independent of military rank. A total of 120 participants participated in the un-manipulated setup. In 31 pairings, LR started in the *at rest* position and the HR started *standing guard* and in 29 pairings *vice versa*.

##### Negotiation phase

When both soldiers had clicked the start-button, soldier A was informed that he was designated to start the experiment *standing guard*. Soldier A was free to choose one of four offers to send to soldier B. These offers were 5, 10, 15, and 20 min and represent the time that soldier A would have to stand guard in this round if B accepted the offer. After receiving the offer from A, soldier B could decide whether to accept or reject it. In case of rejection, soldier A had to send another offer to B. According to the instructions, the participants were aware that each type of duration could be sent only one time per negotiation phase. A duration had to be accepted when no other duration was available in that phase.

##### Guarding phase

When participants agreed upon a duration, a countdown started with the agreed number of min, and soldier A had to stand guard in front of his computer screen during the countdown.

After the countdown reached zero, soldier B was asked via a computer message whether he wished to relieve soldier A. Soldier B’s decision established the basis for the next negotiation phase. If B decided on “No”, a new negotiation phase started again (as described above), with soldier A having to send a new offer. If soldier B decided on “Yes” and relieved soldier A, then soldier B was asked to send an offer to soldier A. These interactions continued until both soldiers had spent a combined total of 40min *standing guard*. Note that the durations of guarding time the participants could choose from during the negotiation phase never exceeded the remaining total time of the experiment. For example, if both soldiers stood guard for a total of 30 min and therefore only 10 min of guard duty remained in the experiment, the soldier who had to send an offer could only choose between offers of 5 and 10 min. The experiment ended after both soldiers spent a combined total of 40 min standing guard. Afterward the soldiers were moved to separate rooms to cool down. Throughout that period they were asked to sit down and relax for 45 min until the end of the experiment. During that time both soldiers viewed images of plants and scenery on a computer screen.

#### Manipulated Setup

The manipulated setup was similar to the un-manipulated one with one exception: the experimenter simulated the reactions of each participant’s opponent. Soldiers were able to make only a few choices of their own, and received pre-defined responses. The beginning of the experiment was exactly the same as in the first setup. After the soldiers read the instructions and clicked the start button, both were informed that they were to start the allocation experiment *standing guard* and that they needed to send an offer to the other soldier, whom they believed to start the allocation experiment *at rest*. In this setup, participants could choose from three durations, 5, 15, and 20 min (adding up to 40) and, contrary to the un-manipulated setup, each duration could be chosen only once in the allocation experiment. The offers the soldiers sent were always accepted (part of the manipulation). However, after the countdown, both soldiers received a message stating that the other soldier decided against relieving them and that they needed to send a new offer. This was continued until each soldier spent 40 min *standing guard*. This experiment was designed to mimic socio negative behavior by the opponent, in that each of the two soldiers was led to believe that his counterpart was *at rest* for 40 min and did not relieve him from *standing guard.* This was done in order to investigate whether socio negative or unfair behavior causes a physiological reaction. Since standing in front of a computer screen itself is not metabolically costly or stressful, physiological effects can be attributed to a feeling of being treated unfairly. The reduced amount of choices, with each choice being available only once in the whole experiment was necessary in order to make all manipulated experiments as alike as possible without giving away the manipulation. A total of 60 participants participated in this setup, 30 HR and 30 LR. For more information regarding the design of the experiments, see Supplementary Figures S1 and S2

#### Collection and Analyses of Saliva Samples

In total, eight saliva samples were taken from each participant from the beginning of an experiment until the end of the cool down phase. All experiments began within a 90 minute time frame between 09.50 AM and 11.20 AM on workdays. The first saliva sample was collected immediately before the experiment started. Additional samples were taken after 10, 20, 30, and 40 min. During the cooldown phase, samples were collected every 15 min. Saliva samples were collected via passive drool using SaliCaps (IBL International, Hamburg, Germany). After the collection the samples were stored immediately on ice for a maximum of two hours until shipment to the laboratory, where they were stored at -20°C.

The samples were analyzed at the endocrine lab of the Department of Behavioral Biology (University of Vienna, Vienna, Austria). T concentrations were measured using IBL Testosterone Saliva enzyme-linked immunosorbent assay (ELISA) kits according to the instructions of the manufacturer (IBL International, Hamburg, Germany; intra-assay-coefficient = 8.6, inter-assay-coefficient = 13.34). C levels were quantified by carrying out an enzyme immunoassay (EIA) in the same lab. The methodological procedures and information about the used antibodies are described elsewhere [Palme and Möstl (1994, 1997) intra-assay-coefficient = 9.2, inter-assay-coefficient = 14.49]. Note that sample size varies between sample-points. This is in part due to participants that did not provide requested saliva samples or did not provide sufficient volume for analysis. For T 1350 samples of 178 soldiers out of 1440 possible samples (8 samples × 180 soldiers) are included in the statistical analysis. The limit of detection for the T assay was 0.002 ng/ml.

For C 1314 samples were analyzed. A total of 393 measurements of C had to be excluded from statistical analysis because the inter-assay coefficient of the standardized pool varied too greatly. C levels from 132 participants are included in the statistical analysis (for exact sample sizes at each sample point see Supplementary Tables S1-S4). The linear mixed-effects models described in Section “Hormone data” yielded the same results whether the measurements resulting from the divergent plates were excluded or not. For a comparison, results including the removed measurements are provided in the Supplementary Table S5. Here however we will discuss only the results from computations where measurements from the divergent ELISA plates were excluded. The limit of detection for the C assay was 0.0033 ng/ml.

### Variable Processing

#### Fair Outcomes in the Un-manipulated Setup

The total amount of time an individual ended up *standing guard* in the course of the allocation experiment was assessed in relation to military rank. Since the results for that variable are complementary for the two players participating in the same allocation experiment, only the results from those individuals that started the experiments *at rest* were used for the analysis. The participant who started the experiment *at rest* had the opportunity of choosing which offers to accept and whether to relieve his opponent or not. Therefore, the participant starting *at rest* never needed to change to the *standing guard* position if he did not choose to do so.

Outcomes after the full 40 min of interaction were categorized into three categories: *Unfair, Fair and Hyperfair*. These categories were defined according to how the 40 min of *standing guard* were shared between the two participants of one session. The categories were defined as *Unfair* for values below 20 min as *Fair* for values of exactly 20 and as *Hyperfair* for values bigger than 20 min. This is based on the assumption of Fehr and Schmidt (1999) that people perceive outcomes as unfair (inequitable) if the outcome deviates from the egalitarian outcome. These categories were used to analyze the *Hyperfair*, *Fair* or *Unfair* outcomes in relation to military rank by calculating a contingency table (Fisher’s exact test, **Table 1**).

**Table 1:** Contingency table of number of *Unfair*, *Fair* and *Hyperfair* outcomes in high ranking (HR) and low ranking (LR) soldiers during the un-manipulated interaction experiment.

	HR	LR
Unfair	16	6
Fair	13	23
Hyperfair	0	2

*The outcome fell into the Fair category if the participant starting at rest spent 50% of the time (20 min) standing guard, the category Hyperfair is used when the participant spent more than 20 min standing guard and Unfair when he stood less than 20 min.*

#### Hormone Data

Both, C and T values were not normally distributed. Data was therefore log10 transformed. Q-Q plots of the used data can be found in the Supplementary Figures S3 and S4. Linear mixed-effects models with the factors *Rank*, *Manipulated*, and *Time* (all eight time points) were applied for log10 transformed C and T levels collected in the study, respectively. There is a significant difference in age between the HR and LR group (Student’s *t*-test, *N*_LR_ = 75, *N*_HR_ = 81, *P* < 0.01) therefore two separate linear mixed-effects models were computed including age instead of rank for C and T levels respectively. Here it is of note, that 16 soldiers did not state their age in the questionnaire that accompanied the experiment. Hence, the sample size for the above mentioned models is smaller than for those that did not use age as a factor. In order to test the effect of the manipulation on the two groups of rank, an additional model was calculated for each the HR and the LR group, using the same factors excluding only the factor *Rank*. The participant’s ID and the time points were used as random factors in all models in order to correct for repeated measurements. Linear mixed-effects models were used because they are reportedly better for datasets including missing values than RM-ANOVAs (Krueger and Tian, 2004).

Student’s t-tests were used to compare log10 transformed T and C levels of HR and LR before the experiments started (T_0_ and C_0_). At this sample point, hormone levels are independent of the two different setups. Therefore, military rank related differences in hormone levels were calculated from samples taken from all participants, regardless of subsequent treatment *(N* C_0_ = 115, *N* T_0_ = 171). For the same time point a correlation between age and C and T levels was calculated using Pearson’s product-moment correlation.

A correlation test between C_40_ and T_40_ with the time a soldier spent *standing guard* in the experiment (Spearman correlation and Spearman’s rho) was calculated for both HR and LR in the un-manipulated setup. The post-interaction-sample point (T_40_, C_40_) was chosen for this analysis because at that sample point all interactions had been concluded. Spearman’s correlation was used because it is a better tool to detect non-linear correlations and there is no reason to assume possible correlations to be linear.

#### T/C Ratios

T/C ratios (T_X_/C_X_ = T_X_/T_X_+C_X_) were calculated for the sample points: T_0_/C_0_ and T_40_/C_40_. T_0_/C_0_ and T_40_/C_40_ ratios were analyzed in participants of different military rank (HR and LR) using *t*-tests.

#### Computation

Student’s *t*-tests, spearman correlations and Fisher’s exact tests were conducted in R (R Core Team, 2015) with α set at 0.05. Linear mixed-effects models were conducted using the R package *nlme* in R (Pinheiro et al., 2015). Graphical Illustrations were created using the *ggplot2* package in R (Wickham, 2009).

## Results

### Behavioral Results - Fairness in the Allocation Experiment

In the un-manipulated setup the average time an individual soldier spent *standing guard* varied according to his status. More precisely, when LR started the experiment *standing guard*, they stood for an average of 24.3 (*SD* = 5.6) min throughout the experiment. In contrast, in the same situation HR *stood guard* for only 21.3 min (*SD* = 4.5) on average. Accordingly, LR spent more time *standing guard* than HR (Mann-Whitney *U* test, *U* = 279, *P* = 0.004, *N* = 60 experiments). The age of the participants did not correlate with the time spent *standing guard* (Spearman’s rank correlation, *P* = 0.9, *rs* = 0.017; *N* = 48).

HR stood for less than 20 min in 16 out of 29 trials (*Unfair*; **Table 1**) in which they started in the *at rest* position. In 13 out of 29, HR ended up standing for 20 min (*Fair*; **Table 1**). HR never stood for more than 20 min when they began the un-manipulated experiment in the *at rest* position.

When a LR started in the *at rest* position, the allocation fell into the *Unfair* category in six out of 31 trials and in the *Fair* category 23 times. Two LR soldiers stood for more than 20 min and ended up in the *Hyperfair* category. Therefore, experiments in which the LR started *at rest* ended in the *Fair* and *Hyperfair* category more often compared to when HR started in that position (**Table 1**, Fishers exact test, *P* = 0.007).

### Hormonal Results

#### Before Experiment

T levels at the beginning of the experiment (T_0_) were marginally significantly different between high rankers and low rankers (Student’s *t*-test, *N*_LR_ = 85; *N*_Hr_ = 86, *P* = 0.056). T was higher in HR than LR (HR mean = 0.49 ng/ml + SE = 0.0539; LR mean = 0.372 ng/ml + SE = 0.0481). C levels at the beginning of the experiment (C_0_) were also marginally significantly different between the ranks (Student’s *t*-test, *N*_LR_ = 51; *N*_HR_ = 64, *P* = 0.187, *P* = 0.08). Age was not significantly correlated with C_0_ levels (*N* = 99, *P* = 0.27, *r* = 0.11) or T_0_ levels (*N* = 152, *P* = 0.74, *r* = -0.03).

No differences in the T/C ratio were found between the ranks before and after the experiment (Supplementary Table S6)

#### Linear Mixed-Effects Models

For C both, Rank (β = 0.179, CI = [0.04,0.32], *P* = 0.015) and Manipulated (β = 0.198, CI = [0.04,0.36], *P* = 0.018) were significant predictors for in the linear mixed-effects model for C levels **Table 2**. In an additional model where the factor Rank was replace by the factor Age, Age was a marginally significant predictor of C levels in the experiment (β = 0.008, CI = [0, 0.02], *P* = 0.063, Supplementary Table S7) Separate analysis of the rank groups showed that for C levels of HR, Manipulated was not a significant predictor (β = 0.127, CI = [-0.08,0.33], *P* = 0.23, Supplementary Table S8). However, Manipulated was a significant predictor of C levels in the LR group (β = 0.283, CI = [0.03, 0.54)], *P* = 0.035, Supplementary Table S9).

**Table 2:** Results from linear mixed-effects model for C levels throughout the experiment.

	β (CI)	*P*-value
(Intercept)	0.385	<0.001
	(0.26,0.51)	
Rank HR (reference LR)	0.179	0.015
	(0.04,0.32)	
Manipulated	0.198	0.018
	(0.04,0.36)	
TimeC10	-0.072	0.059
	(-0.15,0)	
TimeC20	-0.063	0.097
	(-0.14,0.01)	
TimeC30	-0.068	0.075
	(-0.14,0.01)	
TimeC40	-0.08	0.034
	(-0.15,-0.01)	
TimeC55	-0.143	<0.001
	(-0.22,-0.07)	
TimeC70	-0.153	<0.001
	(-0.23,-0.08)	
TimeC85	-0.131	0.001
	(-0.21,-0.05)	

*Factors: rank; military rank, manipulated, effect of the manipulation and Time (TimeC0-TimeC85). β (CI), beta value and 95% confidence interval.*

For T only *Manipulated* was a significant predictor (β = 0.103, CI = [0.01,0.2], *P* = 0.037), and *Rank* was not (**Table 3**). In an additional model where instead of the factor Rank Age was used to predict T values, Age was not a predictor of T levels (β = -0.001, CI = [-0.01,0], *P* = 0.73, Supplementary Table S10). The separate analysis for both groups of rank revealed that *Manipulated* was not a significant predictor for T levels in HR (β = 0.09, CI = [-0.05,0.23], *P* = 0.224, Supplementary Table S11) and a marginally significant predictor for T levels in the LR group (β = 0.116, CI = [-0.01,0.24], *P* = 0.079, Supplementary Table S12).

**Table 3:** Results from linear mixed-effects model for T levels throughout the experiment.

	β (CI)	*P*-value
(Intercept)	-0.602	<0.001
	(-0.68,-0.53)	
Rank HR (reference LR)	0.066	0.155
	(-0.02,0.16)	
Manipulated Yes (reference No)	0.103	0.037
	(0.01,0.2)	
TimeT10	-0.061	0.008
	(-0.11,-0.02)	
TimeT20	-0.085	<0.001
	(-0.13,-0.04)	
TimeT30	-0.123	<0.001
	(-0.17,-0.08)	
TimeT40	-0.085	<0.001
	(-0.13,-0.04)	
TimeT55	-0.051	0.028
	(-0.1,-0.01)	
TimeT70	-0.09	<0.001
	(-0.14,-0.04)	
TimeT85	-0.071	0.002
	(-0.12,-0.03)	

*Factors: rank; military rank, manipulated, effect of the manipulation and Time (TimeT0-TimeT85). β (CI), beta value and 95% confidence interval.*

**FIGURE 1:**
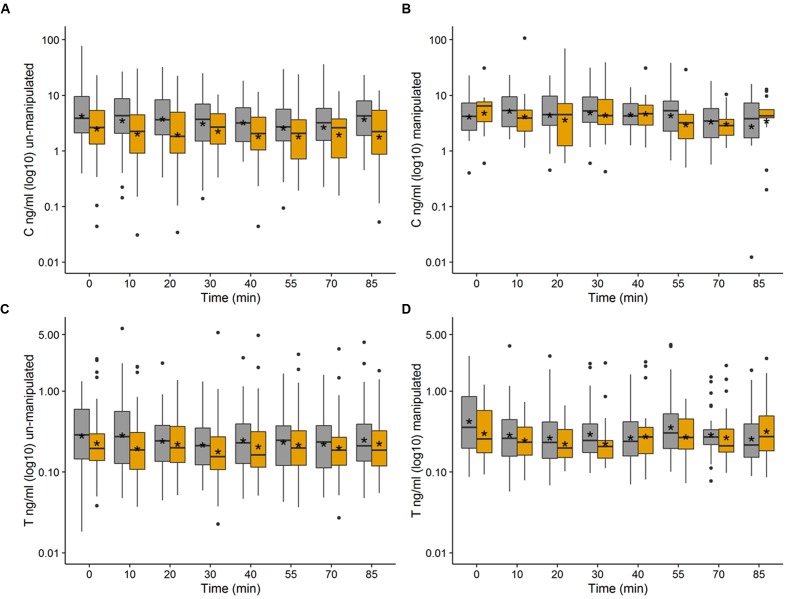
**(A-D)** Boxplots of log10 transformed C = cortisol **(A,B)** and T = testosterone levels **(C,D)** during the un-manipulated **(A,C)** and manipulated **(B,D)** setup in an interaction experiment testing high-ranking soldiers (HR, gray boxes) against low-ranking soldiers (LR, orange boxes), an asterisk within each box marks the mean-value.

A boxplot of the log 10 transformed data has been used to plot C and T levels in the un-manipulated (**Figures 1A,C**) and manipulated experiment (**Figures 1B,D**). A table presenting the mean, standard deviation, and confidence interval for all time points and groups can be found in the Supplementary Tables S1-S4.

Correlations between T0, C0, or T/C0 and the time spent standing guard did not yield any significant results (Supplementary Table S13).

## Discussion

The present study investigated how social rank influences fairness in human interactions and hormone levels. Using a novel allocation task we showed that military rank has an effect on the fairness of the allocation. Physiologically we showed that warrant officers (HR) had higher levels of cortisol during the experiment than enlisted men (LR).

In this study, soldiers were used because they are part of a military cohort in which social rank was clearly defined and easily and accurately detectable. The Austrian Armed Forces provided us with two groups of rank: warrant officers and enlisted men. Soldiers of these groups were chosen because they are clearly distinct from each other as far as military rank is concerned but differences in income and education are minimal. The experiment did not use money as an incentive. This set it apart from most economic games (Camerer and Thaler, 1995; Cameron, 1999). The incentive in the experiment was the amount of time a participant spent *standing guard* during the session. Note that in our experiment the soldier who started in the *at rest* position decided which offers he accepted from his opponent and whether to relieve him from the *standing guard* position or not. The participant starting *at rest* had control over the game and never needed to change to the *standing guard* position at all.

During the experiment, HR spent less time in the *standing guard* position than LR. Accordingly, more experiments ended in the *Unfair* category when HR started the experiment *at rest*. Such a result raises the question why elevated rank should influence the results in this way although no acquisition of resources was involved. Piff et al. (2012) argue that unethical or unfair behavior in people of higher status is partially caused by a more favorable attitude towards greed. This explanation may not be applicable to the present study because no economic incentive was involved and participants had nothing to gain from the game’s outcome. Another study, however, found lower SES to be associated with more generous, charitable, trusting, and helpful behavior than higher SES (Piff et al., 2010). LR granting privileges to HR could explain why HR spent less time *standing guard* in comparison to LR. It is also possible that HR, as a consequence of their elevated rank, felt more entitled to be in the more advantageous position than the LR (Major, 1994; O’Brien and Major, 2009). This allowed them “naturally” to sit while their LR opponents had to stand.

Our investigation on rank and hormone levels before the experiment indicate elevated T levels in HR compared to LR soldiers. This trend is in line with the theory of a positive relationship between status and T in men (reviewed in Mazur and Booth, 1998). However, the marginal difference between T levels in HR and LR did not persist in the linear mixed-effects model. Additional analysis would be necessary to evaluate whether military rank correlates with basal T levels in soldiers. Fairness has been suggested to be correlated with T levels or the T/C ratio (Burnham, 2007; Eisenegger et al., 2010). Here, we found no correlation between T levels or T/C ratio and time spent *standing guard*, although in our study military rank was marginally correlated with T_0_ and C_0_ levels and is predictive of the fairness of the outcome of the un-manipulated experiment. Our results therefore do not indicate an effect of hormone levels on fairness behavior. The unfair treatment simulated in the manipulated version of the allocation experiment had a marginally positive effect on T levels in LR but not in HR soldiers. However, given the somewhat weak effects of the manipulation even in the LR group we cannot draw any conclusions regarding the effects of rank and fairness on T levels in soldiers.

Our result, however, provide us with clear evidence on increased C levels in HR compared to LR. This association of C levels and military rank persisted throughout the experiment. The difference between the groups of rank was marginal before the experiment but became even more pronounced during the experiment. Besides rank the unfair treatment the soldiers experienced in the manipulated set-up was associated with increased C levels. However, when the groups of rank were analyzed separately the effect of unfair treatment on C levels persisted only in the LR group. The above mentioned findings lead us to the assumption that HR felt challenged by the situation of facing a LR outside of common military routine, whereas LR only reacted when treated unfairly. The military is a very rigid hierarchy. Rapid, unpredictable changes in the rank structure are almost impossible. This hierarchy is more stable than those observed in other primates. Our findings seem to be in dissent with previous findings on non-human primates reporting increased basal levels of glucocorticoids in low compared to higher ranked individuals living in stable hierarchies that are maintained non-physically (reviewed in Sapolsky, 2005). They rather support other studies reporting, higher C levels in alpha males (Barrett et al., 2002; Girard-Buttoz et al., 2014). In these studies higher C levels in high ranked individuals were explained by greater metaboloic costs associated with prolonged courtships and mate-guarding activities. In our experiment metabolic costs cannot have caused the differences in C levels, since *standing guard* in front of a computer screen is not metabollically costly for soldiers. Furthermore, the group that exhibited higher C levels (HR) spent less time *standing guard* than the group with lower C levels (LR).

We therefore conclude, that interacting with a member of the same cohort, but outside of the very strict hierarchical rules of that cohort, was more challenging to the higher ranking participants than the lower ranking ones. We assume, that this is caused by higher ranking soldier experiencing a threat to their authority and status by the interaction.

The induced unfair treatment during the manipulated set-up had an effect on C levels, with C levels being higher than in the un-manipulated experiment. Therefore, it would stand to reason that “naturally” occurring unfair allocations in the un-manipulated setup would also correlate with C levels. However, we did not find such an effect. This could be explained by the severity and frequency of the mimicked behavior in the manipulated setup. Here, *standing guard* for all participants lasted 40 min, with all reliefs being rejected. The same was true for only three out of 120 participants in the un-manipulated setup.

As has been visualized in **Figures 1A–D** (also see Supplementary Tables S1-S4) the individual variability of hormone levels is quite high in both setups, but especially in the manipulated experiment. Here the variability appears to be increased in testosterone in HR and in cortisol in LR. One explanation for this would be that HR more often react to unfair treatment in a rise in testosterone, whereas LR react with increases in cortisol. The variation could then be explained by individuals in each group that are less responsive than others. This would be in line with testosterone frequently being associated with dominance and status seeking behavior (Mazur and Booth, 1998; Eisenegger et al., 2011). Hence HR might feel challenged in their status by being treated unfairly by a LR. However, with the available data and given the high inter-individual variability we cannot draw reliable conclusions in this regard.

In this study, the HR group was older than the LR group by an average of approximately 12 years. Age is therefore a possible confounding variable in regard to rank. Van Cauter et al. (1996) found age to affect the circadian rhythmicity of plasma C levels in both sexes. In contrast, Wust et al. (2000) did not find a significant correlation between C awakening response and age. In our study neither C nor T levels before the experiment (C_0_, T_0_) were correlated with age. Although, there was a trend indicating age to have an effect on C levels during the experiment, the effects of rank on C levels appear to be more robust than those of age. Hence findings of the present study suggest that rank is a better predictor of C levels in soldiers than age.

To summarize, the main findings of our study are that HR had higher C levels during and after the interaction than their LR counterparts and that results from the allocation experiment fell into the *Unfair* category more often when HR were in control at the beginning of the interaction. We assume that HR may have felt entitled to be in the more desirable position and felt a greater need to “win” the allocation experiment in order to support their status. An alternative explanation is that LR automatically deferred to HR in the experiment. Whether rank was actively being pulled in our experiment cannot be ascertained. Our data do, however, support the conclusion that military rank was an important factor: it modifies how long a soldier had to stand guard as well as his endocrine status in the course of the experiment. To study the cause of these effects further studies are required. For the purpose of studying the effect of status on fairness it would be intriguing to use the structure of military hierarchies in economic laboratory experiments, such as the dictator (Kahneman et al., 1986) and ultimatum game (Güth et al., 1982). Here it would be of interest to apply personality questionnaires and other techniques to investigate the variability in physiological reactions, especially as a response to fair and unfair behavior.

## Author Contributions

BS contributed to the research design, data collection in the field, data analyses, and writing of the manuscript. LP contributed to data analyses, and writing of the manuscript. BW contributed and commented on the research design, writing of the manuscript, and contributed material for analysis.

## Conflict of Interest Statement

The authors declare that the research was conducted in the absence of any commercial or financial relationships that could be construed as a potential conflict of interest.
