# Corrigendum: Pulling Rank: Military Rank Affects Hormone Levels and Fairness in an Allocation Experiment

**DOI:** 10.3389/fpsyg.2017.00955

**Published:** 2017-06-08

**Authors:** Benjamin Siart, Lena S. Pflüger, Bernard Wallner

**Affiliations:** ^1^Department of Anthropology, University of ViennaVienna, Austria; ^2^Department of Behavioural Biology, University of ViennaVienna, Austria

**Keywords:** hierarchy, status, military rank, cortisol, testosterone, allocation experiment, social

In the original article, there was an error. In the Acknowledgment section, a wrong name was used.

A correction has been made to the acknowledgment section, [Paragraph 1]:

We thank all participating soldiers and helpers on site. We thank the Austrian Armed Forces especially (Vzlt Greiß, Olt Bachmann, Dr. Martin Jancuska) and the Austrian Federal Ministry of Defense and Sports Science, Research and Development Division (Brigadier Hofmeister, Adelheid Obwaller) for making this study possible. We are grateful to Elisabeth Pschernig and Samy El- Makarem for their support in the ELISA laboratory. We are grateful to Martin Fieder and Markus Koppensteiner for their council in matters of statistics and proof-reading this manuscript and to Peter Kuttenberger for his contribution to the programing of the experiment. This article was supported by the Open Access Publishing Fund of the University of Vienna.

The authors apologize for this error and state that this does not change the scientific conclusions of the article in any way.

## Conflict of interest statement

The authors declare that the research was conducted in the absence of any commercial or financial relationships that could be construed as a potential conflict of interest.

