# Supplementary material for: Pulling Rank: Military Rank Affects Hormone Levels and Fairness in an Allocation Experiment
Source: Front Psychol. 2016 Nov 11;7:1750. doi: 10.3389/fpsyg.2016.01750 (PMC5104734; doi:10.3389/fpsyg.2016.01750)
Supplement: Supplementary file 1 [file Data_Sheet_1.doc]

## Supplementary Figures


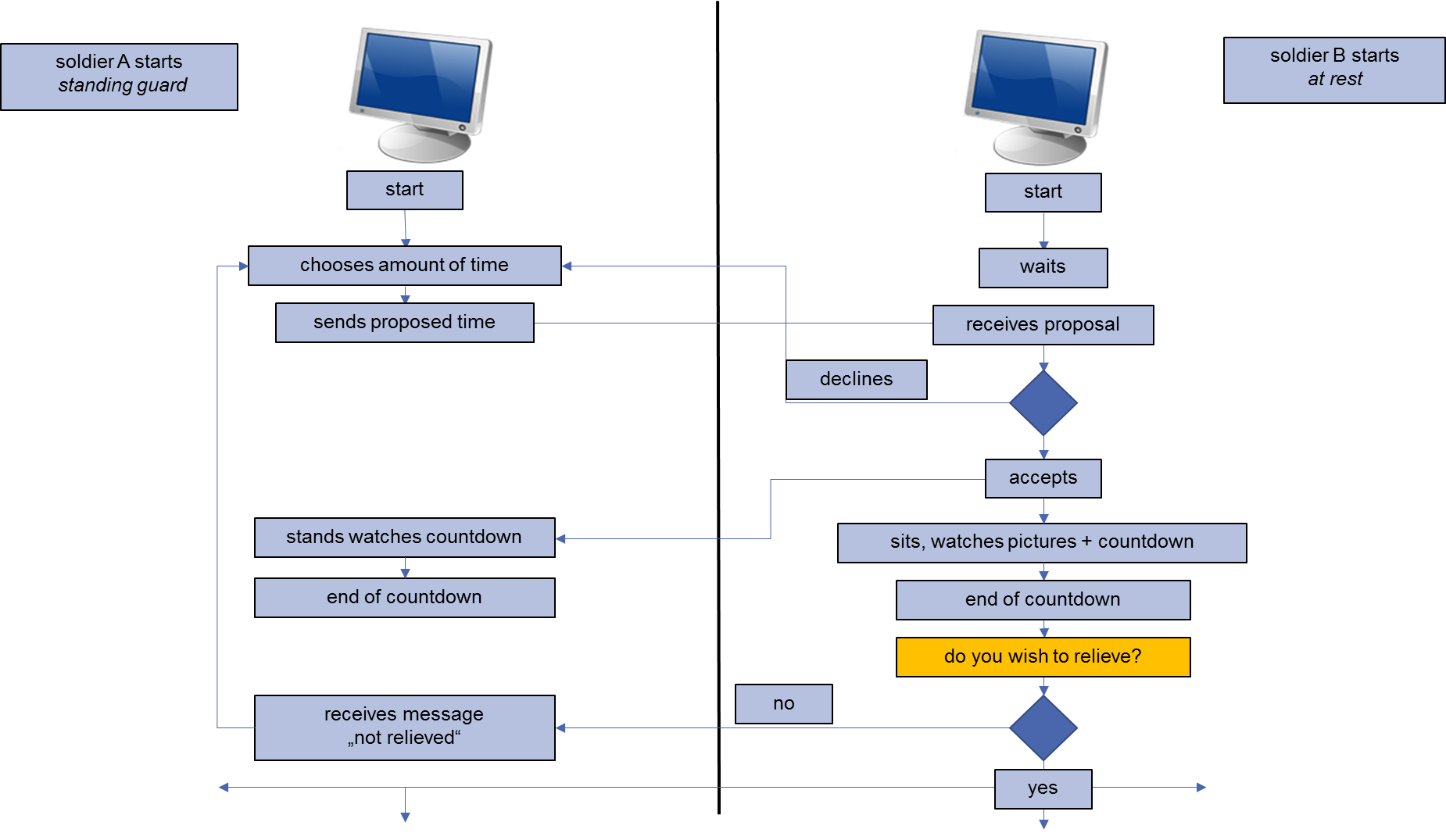


Supplementary Figure 1) un-manipulated setup in interaction experiment


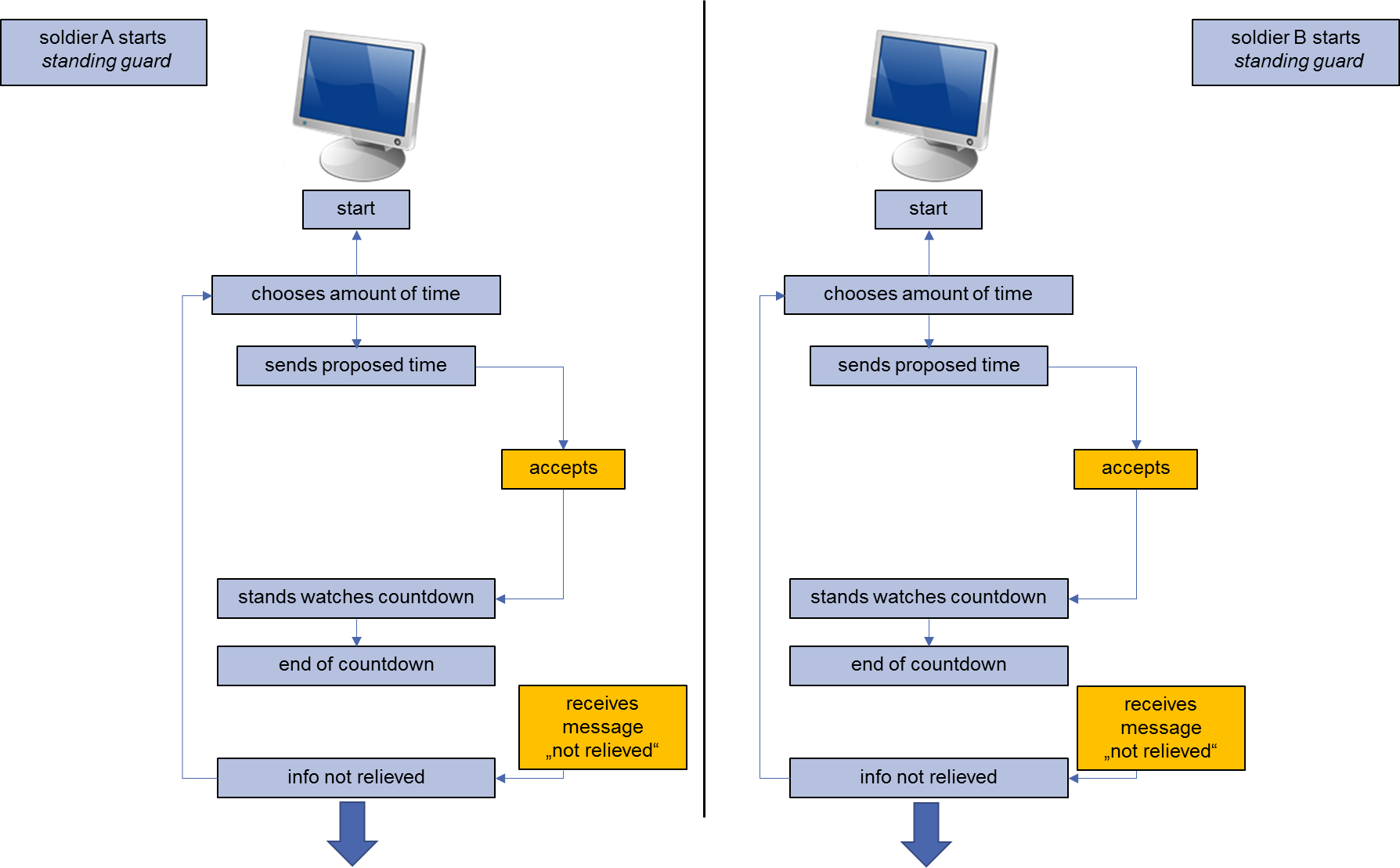


Supplementary Figure 2) manipulated setup of interaction experiment


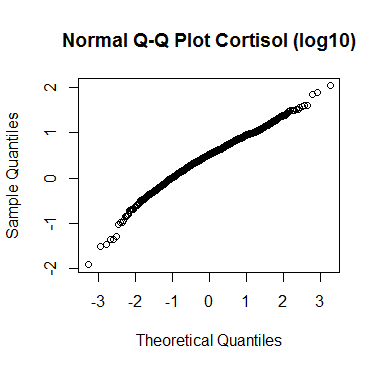


Supplementary Figure3) Q-Q plot for log10 transformed cortisol values.


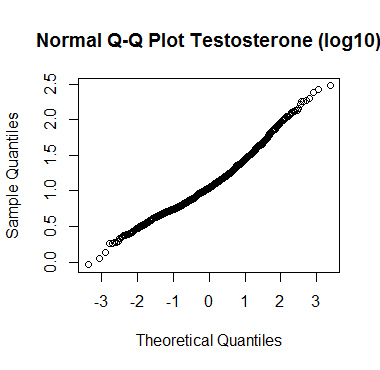


Supplementary Figure 4) Q-Q plot for log10 transformed testosterone values.

## Supplementary Tables

Supplementary Table 1) Cortisol (C) levels 0-85 min after the start of the experiment (C_0_-C_85_) in HR group in the course of the manipulated and un-manipulated experiment (Manipulated = Yes or No). Mean, N= sample size, sd = standard deviation, se = standart error, ci = confidence interval (95%)

| Time | Rank | Manipulated | N | Mean Cortisol (ng/ml) | sd | se | ci |
| --- | --- | --- | --- | --- | --- | --- | --- |
| C0 | HR | No | 47 | 7.95 | 12.48 | 1.82 | 3.66 |
| C0 | HR | Yes | 17 | 6.45 | 6.43 | 1.56 | 3.30 |
| C10 | HR | No | 47 | 5.72 | 5.42 | 0.79 | 1.59 |
| C10 | HR | Yes | 18 | 7.06 | 5.88 | 1.39 | 2.92 |
| C20 | HR | No | 46 | 5.88 | 6.09 | 0.90 | 1.81 |
| C20 | HR | Yes | 18 | 6.78 | 5.92 | 1.39 | 2.94 |
| C30 | HR | No | 45 | 5.50 | 5.84 | 0.87 | 1.76 |
| C30 | HR | Yes | 18 | 7.40 | 7.48 | 1.76 | 3.72 |
| C40 | HR | No | 48 | 4.75 | 4.21 | 0.61 | 1.22 |
| C40 | HR | Yes | 18 | 5.46 | 3.52 | 0.83 | 1.75 |
| C55 | HR | No | 48 | 4.33 | 4.92 | 0.71 | 1.43 |
| C55 | HR | Yes | 17 | 7.25 | 8.89 | 2.16 | 4.57 |
| C70 | HR | No | 40 | 4.42 | 5.82 | 0.92 | 1.86 |
| C70 | HR | Yes | 18 | 4.91 | 4.67 | 1.10 | 2.32 |
| C85 | HR | No | 37 | 5.41 | 4.68 | 0.77 | 1.56 |
| C85 | HR | Yes | 17 | 4.93 | 4.26 | 1.03 | 2.19 |

Supplementary Table 2) Cortisol (C) levels 0-85 min after the start of the experiment (C_0_-C_85_) in LR group in the course of the manipulated and un-manipulated experiment (Manipulated = Yes or No). Mean, N= sample size, sd = standard deviation, se = standart error, ci = confidence interval (95%)

| Time | rank | Manipulated | N | Mean Cortisol (ng/ml) | sd | se | ci |
| --- | --- | --- | --- | --- | --- | --- | --- |
| C0 | LR | No | 40 | 4.64 | 5.26 | 0.83 | 1.68 |
| C0 | LR | Yes | 11 | 7.41 | 8.23 | 2.48 | 5.53 |
| C10 | LR | No | 41 | 4.82 | 7.41 | 1.16 | 2.34 |
| C10 | LR | Yes | 14 | 11.24 | 27.49 | 7.35 | 15.87 |
| C20 | LR | No | 43 | 4.07 | 5.34 | 0.81 | 1.64 |
| C20 | LR | Yes | 14 | 8.88 | 17.69 | 4.73 | 10.22 |
| C30 | LR | No | 41 | 3.27 | 2.76 | 0.43 | 0.87 |
| C30 | LR | Yes | 14 | 7.57 | 9.81 | 2.62 | 5.66 |
| C40 | LR | No | 43 | 2.93 | 2.70 | 0.41 | 0.83 |
| C40 | LR | Yes | 12 | 6.94 | 8.04 | 2.32 | 5.11 |
| C55 | LR | No | 41 | 3.35 | 4.46 | 0.70 | 1.41 |
| C55 | LR | Yes | 14 | 4.90 | 7.13 | 1.90 | 4.11 |
| C70 | LR | No | 37 | 3.21 | 3.08 | 0.51 | 1.03 |
| C70 | LR | Yes | 11 | 3.87 | 3.06 | 0.92 | 2.06 |
| C85 | LR | No | 33 | 3.37 | 3.10 | 0.54 | 1.10 |
| C85 | LR | Yes | 13 | 5.31 | 3.89 | 1.08 | 2.35 |

Supplementary Table 3) Testosterone (T) levels 0-85 min after the start of the experiment (T_0_-T_85_) in LR group in the course of the manipulated and un-manipulated experiment (Manipulated = Yes or No). Mean. N= sample size. sd = standard deviation. se = standart error. ci = confidence interval (95%).

| Time | rank | Manipulated | N | Mean Testosterone (ng/ml) | sd | se | ci |
| --- | --- | --- | --- | --- | --- | --- | --- |
| T0 | LR | No | 58 | 0.36 | 0.50 | 0.07 | 0.13 |
| T0 | LR | Yes | 27 | 0.40 | 0.30 | 0.06 | 0.12 |
| T10 | LR | No | 60 | 0.30 | 0.40 | 0.05 | 0.10 |
| T10 | LR | Yes | 26 | 0.29 | 0.17 | 0.03 | 0.07 |
| T20 | LR | No | 59 | 0.30 | 0.29 | 0.04 | 0.08 |
| T20 | LR | Yes | 26 | 0.26 | 0.16 | 0.03 | 0.06 |
| T30 | LR | No | 57 | 0.31 | 0.70 | 0.09 | 0.18 |
| T30 | LR | Yes | 27 | 0.31 | 0.41 | 0.08 | 0.16 |
| T40 | LR | No | 59 | 0.36 | 0.68 | 0.09 | 0.18 |
| T40 | LR | Yes | 28 | 0.43 | 0.55 | 0.10 | 0.21 |
| T55 | LR | No | 59 | 0.34 | 0.46 | 0.06 | 0.12 |
| T55 | LR | Yes | 26 | 0.32 | 0.18 | 0.04 | 0.07 |
| T70 | LR | No | 58 | 0.31 | 0.47 | 0.06 | 0.12 |
| T70 | LR | Yes | 27 | 0.38 | 0.44 | 0.09 | 0.18 |
| T85 | LR | No | 55 | 0.33 | 0.36 | 0.05 | 0.10 |
| T85 | LR | Yes | 25 | 0.47 | 0.54 | 0.11 | 0.22 |

Supplementary Table 4) Testosterone (T) levels 0-85 min after the start of the experiment (T_0_-T_85_) in HR group in the course of the manipulated and un-manipulated experiment (Manipulated = Yes or No). Mean. N= sample size. sd = standard deviation. se = standart error. ci = confidence interval (95%).

| Time | rank | Manipulated | N | Mean Testosterone (ng/ml) | sd | se | ci |
| --- | --- | --- | --- | --- | --- | --- | --- |
| T0 | HR | No | 57 | 0.40 | 0.30 | 0.04 | 0.08 |
| T0 | HR | Yes | 29 | 0.69 | 0.72 | 0.13 | 0.27 |
| T10 | HR | No | 57 | 0.50 | 0.84 | 0.11 | 0.22 |
| T10 | HR | Yes | 28 | 0.46 | 0.67 | 0.13 | 0.26 |
| T20 | HR | No | 56 | 0.33 | 0.33 | 0.04 | 0.09 |
| T20 | HR | Yes | 29 | 0.43 | 0.58 | 0.11 | 0.22 |
| T30 | HR | No | 55 | 0.29 | 0.27 | 0.04 | 0.07 |
| T30 | HR | Yes | 28 | 0.44 | 0.52 | 0.10 | 0.20 |
| T40 | HR | No | 59 | 0.36 | 0.40 | 0.05 | 0.10 |
| T40 | HR | Yes | 29 | 0.36 | 0.35 | 0.07 | 0.13 |
| T55 | HR | No | 55 | 0.33 | 0.31 | 0.04 | 0.08 |
| T55 | HR | Yes | 29 | 0.62 | 0.91 | 0.17 | 0.35 |
| T70 | HR | No | 55 | 0.32 | 0.32 | 0.04 | 0.09 |
| T70 | HR | Yes | 28 | 0.38 | 0.35 | 0.07 | 0.13 |
| T85 | HR | No | 53 | 0.44 | 0.65 | 0.09 | 0.18 |
| T85 | HR | Yes | 29 | 0.36 | 0.39 | 0.07 | 0.15 |

Supplementary Table 5) Results from linear mixed-effects model for C levels throughout the experiment including C levels excluded due to divergent ELISA plates

|  | Beta.CI | P.value |
| --- | --- | --- |
| (Intercept) | 0.395 | <0.001 |
|  | (0.29,0.5) |  |
| rank HR  (reference LR) | 0.128 | 0.041 |
|  | (0.01,0.25) |  |
| Manipulated Yes  (reference No) | 0.163 | 0.015 |
|  | (0.03,0.29) |  |
| TimeC10 | -0.04 | 0.204 |
|  | (-0.1,0.02) |  |
| TimeC20 | -0.045 | 0.153 |
|  | (-0.11,0.02) |  |
| TimeC30 | -0.043 | 0.176 |
|  | (-0.1,0.02) |  |
| TimeC40 | -0.07 | 0.025 |
|  | (-0.13,-0.01) |  |
| TimeC55 | -0.095 | 0.002 |
|  | (-0.16,-0.03) |  |
| TimeC70 | -0.15 | <0.001 |
|  | (-0.21,-0.09) |  |
| TimeC85 | -0.084 | 0.012 |
|  | (-0.15,-0.02) |  |

Factors: rank; military rank, manipulated, effect of the manipulation and Time (TimeC0-TimeC85). Beta.CI, beta value and 95% confidence interval.

Supplementary Table 6) results from Mann-Whitney *U* test and student’s-test (using log10 transformed values) between military rank (high rank = HR, low rank = LR) and the testosterone to cortisol ratio before (T/C_0_) and after (T/C_40_) 40 min of interaction experiment in the manipulated and un-manipulated treatment. *N* = sample size.

| un-manipulated | T/C_0_ | T/C_40_ | manipulated | T/C_0_ | T/C_40_ |
| --- | --- | --- | --- | --- | --- |
| *N*_LR_ | 39 | 42 | *N*_LR_ | 10 | 12 |
| *N*_HR_ | 45 | 48 | *N*_HR_ | 17 | 18 |
| Mann-Whitney *U* | 792 | 876 | Mann-Whitney *U* | 76 | 100 |
| *P* _Mann-Whitney_ *_U_* | 0.443 | 0.286 | *P* _Mann-Whitney_ *_U_* | 0.651 | 0.735 |
| *P _t-test_* | 0.271 | 0.212 | *P _t-test_* | 0.642 | 0.764 |

Supplementary Table 7) Results from linear mixed-effects model for C levels throughout the experiment including Age instead of Rank

|  | Beta.CI | P.value |
| --- | --- | --- |
| (Intercept) | 0.28 | 0.029 |
|  | (0.03,0.53) |  |
| Age | 0.008 | 0.063 |
|  | (0,0.02) |  |
| ManipulatedYes | 0.152 | 0.079 |
|  | (-0.02,0.32) |  |
| TimeC10 | -0.057 | 0.166 |
|  | (-0.14,0.02) |  |
| TimeC20 | -0.031 | 0.452 |
|  | (-0.11,0.05) |  |
| TimeC30 | -0.052 | 0.211 |
|  | (-0.13,0.03) |  |
| TimeC40 | -0.087 | 0.033 |
|  | (-0.17,-0.01) |  |
| TimeC55 | -0.127 | 0.002 |
|  | (-0.21,-0.05) |  |
| TimeC70 | -0.151 | <0.001 |
|  | (-0.23,-0.07) |  |
| TimeC85 | -0.113 | 0.009 |
|  | (-0.2,-0.03) |  |

Factors: Age, manipulated, effect of the manipulation and Time (TimeC0-TimeC85). Beta.CI, beta value and 95% confidence interval.

Supplementary Table 8) Results from linear mixed-effects model for C levels throughout the experiment in HR group

| HR | Beta.CI | P.value |
| --- | --- | --- |
| (Intercept) | 0.577 | <0.001 |
|  | (0.45,0.7) |  |
| Manipulated Yes (reference No) | 0.127 | 0.23 |
|  | (-0.08,0.33) |  |
| TimeC10 | -0.033 | 0.514 |
|  | (-0.13,0.07) |  |
| TimeC20 | -0.028 | 0.585 |
|  | (-0.13,0.07) |  |
| TimeC30 | -0.072 | 0.156 |
|  | (-0.17,0.03) |  |
| TimeC40 | -0.077 | 0.127 |
|  | (-0.18,0.02) |  |
| TimeC55 | -0.136 | 0.008 |
|  | (-0.24,-0.04) |  |
| TimeC70 | -0.188 | <0.001 |
|  | (-0.29,-0.09) |  |
| TimeC85 | -0.138 | 0.01 |
|  | (-0.24,-0.03) |  |

Factors: manipulated, effect of the manipulation and Time (TimeC0-TimeC85). Beta.CI, beta value and 95% confidence interval.

Supplementary Table 9) Results from linear mixed-effects model for C levels throughout the experiment in LR group

| LR | Beta.CI | P.value |
| --- | --- | --- |
| (Intercept) | 0.372 | <0.001 |
|  | (0.22,0.52) |  |
| Manipulated Yes (reference No) | 0.283 | 0.035 |
|  | (0.03,0.54) |  |
| TimeC10 | -0.121 | 0.036 |
|  | (-0.23,-0.01) |  |
| TimeC20 | -0.104 | 0.067 |
|  | (-0.21,0.01) |  |
| TimeC30 | -0.065 | 0.257 |
|  | (-0.18,0.05) |  |
| TimeC40 | -0.085 | 0.14 |
|  | (-0.2,0.03) |  |
| TimeC55 | -0.155 | 0.007 |
|  | (-0.27,-0.04) |  |
| TimeC70 | -0.112 | 0.059 |
|  | (-0.23,0) |  |
| TimeC85 | -0.122 | 0.043 |
|  | (-0.24,0) |  |

Factors: manipulated, effect of the manipulation and Time (TimeC0-TimeC85). Beta.CI, beta value and 95% confidence interval.

Supplementary Table 10) Results from linear mixed-effects model for T levels throughout the experiment including Age rinstead of Rank

|  | Beta.CI | P.value |
| --- | --- | --- |
| (Intercept) | -0.529 | <0.001 |
|  | (-0.68,-0.38) |  |
| Age | -0.001 | 0.725 |
|  | (-0.01,0) |  |
| ManipulatedYes | 0.1 | 0.048 |
|  | (0,0.2) |  |
| TimeT10 | -0.076 | 0.001 |
|  | (-0.12,-0.03) |  |
| TimeT20 | -0.097 | <0.001 |
|  | (-0.14,-0.05) |  |
| TimeT30 | -0.132 | <0.001 |
|  | (-0.18,-0.08) |  |
| TimeT40 | -0.1 | <0.001 |
|  | (-0.15,-0.05) |  |
| TimeT55 | -0.066 | 0.006 |
|  | (-0.11,-0.02) |  |
| TimeT70 | -0.105 | <0.001 |
|  | (-0.15,-0.06) |  |
| TimeT85 | -0.078 | 0.001 |
|  | (-0.13,-0.03) |  |

Factors: Age, manipulated, effect of the manipulation and Time (TimeT0-TimeT85). Beta.CI, beta value and 95% confidence interval.

Supplementary Table 11) Results from linear mixed-effects model for T levels throughout the experiment in HR group

| HR | Beta.CI | P.value |
| --- | --- | --- |
| (Intercept) | -0.516 | <0.001 |
|  | (-0.61,-0.42) |  |
| Manipulated Yes (reference No) | 0.09 | 0,224 |
|  | (-0.05,0.23) |  |
| TimeT10 | -0.04 | 0,205 |
|  | (-0.1,0.02) |  |
| TimeT20 | -0.112 | <0.001 |
|  | (-0.17,-0.05) |  |
| TimeT30 | -0.126 | <0.001 |
|  | (-0.19,-0.06) |  |
| TimeT40 | -0.107 | 0,001 |
|  | (-0.17,-0.05) |  |
| TimeT55 | -0.073 | 0,021 |
|  | (-0.13,-0.01) |  |
| TimeT70 | -0.115 | <0.001 |
|  | (-0.18,-0.05) |  |
| TimeT85 | -0.118 | <0.001 |
|  | (-0.18,-0.06) |  |

Factors: manipulated, effect of the manipulation and Time (TimeT0-TimeT85). Beta.CI, beta value and 95% confidence interval.

Supplementary Table 12) Results from linear mixed-effects model for T levels throughout the experiment in LR group

| LR | Beta.CI | P.value |
| --- | --- | --- |
| (Intercept) | -0.622 | <0.001 |
|  | (-0.71,-0.54) |  |
| Manipulated Yes (reference No) | 0.116 | 0.079 |
|  | (-0.01,0.24) |  |
| TimeT10 | -0.081 | 0.016 |
|  | (-0.15,-0.02) |  |
| TimeT20 | -0.058 | 0.087 |
|  | (-0.12,0.01) |  |
| TimeT30 | -0.119 | <0.001 |
|  | (-0.18,-0.05) |  |
| TimeT40 | -0.061 | 0.067 |
|  | (-0.13,0) |  |
| TimeT55 | -0.028 | 0.398 |
|  | (-0.09,0.04) |  |
| TimeT70 | -0.065 | 0.054 |
|  | (-0.13,0) |  |
| TimeT85 | -0.023 | 0.509 |
|  | (-0.09,0.04) |  |

Factors: Manipulated, effect of the manipulation and Time (TimeT0-TimeT85). Beta.CI, beta value and 95% confidence interval.

Supplementary Table 13) results from spearman correlation between cortisol (C) and testosterone (T) levels and their ratio (T/C) and the time spent standing guard during the experiment for high ranking (HR) and low ranking (LR) soldiers before (_0_) and after 40 min (_40_) of interaction experiment. *N* = sample size. *r_s_* = Spearman’s rho.

| LR | C_0_ | C_40_ | T_0_ | T_40_ | T/C_0_ | T/C_40_ |
| --- | --- | --- | --- | --- | --- | --- |
| *N* | 40 | 43 | 58 | 59 | 39 | 42 |
| *r_s_* | 0.11 | -0.0003 | -0.07 | 0.05 | -0.19 | 0.05 |
| *P* | 0.49 | 0.99 | 0.59 | 0.69 | 0.26 | 0.74 |
|  |  |  |  |  |  |  |
| HR | C_0_ | C_40_ | T_0_ | T_40_ | T/C_0_ | T/C_40_ |
| *N* | 47 | 48 | 57 | 59 | 45 | 48 |
| *r_s_* | -0.161 | -0.132 | 0.02 | 0.04 | 0.18 | 0.12 |
| *P* | 0.285 | 0.37 | 0.88 | 0.75 | 0.24 | 0.42 |
